# Hybrid single staged treatment of coronary arteries and aorto-iliac obstruction disease: a case report

**DOI:** 10.1186/s13019-022-01797-8

**Published:** 2022-03-21

**Authors:** Keiichiro Kasama, Yasuko Uranaka, Hiroto Tomita, Atsushi Matsumoto, Shinichi Suzuki

**Affiliations:** 1 Department of Cardiovascular Surgery, Yokohama Municipal Citizens Hospital, 1-1 Mitsuzawa Nishicho, Kanagawa-ku, Yokohama, 221-0855 Japan; 2 Department of Surgery, Yokohama City University Graduate of Medicine, Yokohama, Japan

**Keywords:** Coronary artery bypass grafting, Bilateral iliac artery disease, Iliac artery obstruction

## Abstract

**Background:**

If the internal thoracic artery is a collateral circulation to the lower extremities, careful consideration should be given to its use when coronary artery bypass grafting is required. We report a case of CABG with bilateral common iliac artery lesions and collateral circulation from the bilateral ITAs on the peripheral side.

**Case presentation:**

A 58-year-old man was admitted to our department with claudication and dyspnea upon exertion. He was diagnosed with right common iliac artery obstruction and 90% stenosis of the left common iliac artery. Coronary angiography revealed three-vessel disease with 50% stenosis of the left main trunk. The bilateral ITA showed a rich collateral flow to the lower extremities. Hybrid single staged repair with percutaneous transluminal angioplasty for the left iliac lesion was performed, followed by off-pump coronary artery bypass grafting (CABG) and femoro-femoral crossover bypass. Postoperative angiography revealed that all grafts were patent. The postoperative course was uneventful, except that the patient’s creatinine kinase level increased to 7177 U/L on postoperative day 1.

**Conclusion:**

To treat coronary artery disease with peripheral artery disease, especially those with iliac artery occlusion lesions with collateral circulation from the ITA, not only graft selection but also the treatment strategies for peripheral lesions are considered extremely important. Hybrid single staged coronary and lower limb artery revascularization could be safely achieved by multidisciplinary team strategies.

## Background

Coronary artery bypass grafting (CABG) is common in cases with peripheral artery disease [1]. Among the cases, collateral circulation from the internal thoracic artery (ITA) plays an important role in cases of occlusive disease of the aorto-iliac artery region; and graft selection for the coronary artery bypass graft is controversial, in which the appropriate strategy is not well discussed. We report a case of CABG with bilateral common iliac artery lesions and collateral circulation from the bilateral ITAs on the peripheral side.

## Case presentation

We report a case of a 58-year-old male with peripheral artery disease. Although he had a 10-year history of intermittent claudication, he had not visited a hospital for consultation previously. When he experienced a stroke and was admitted to our hospital, an MRI revealed left carotid stenosis, and stenting was performed. During admission, arteriosclerosis obliterans (ASO) was detected. The ankle brachial pressure index (ABI) was 0.61 in his right limb and 0.76 in his left limb. He also had a history of depression, alcoholism, Chronic obstructive pulmonary disease (FEV1.0 was 45.7%, FEV1.0 predicted was 54.8%), hypertension, and chronic renal failure.

**Fig. 1:**
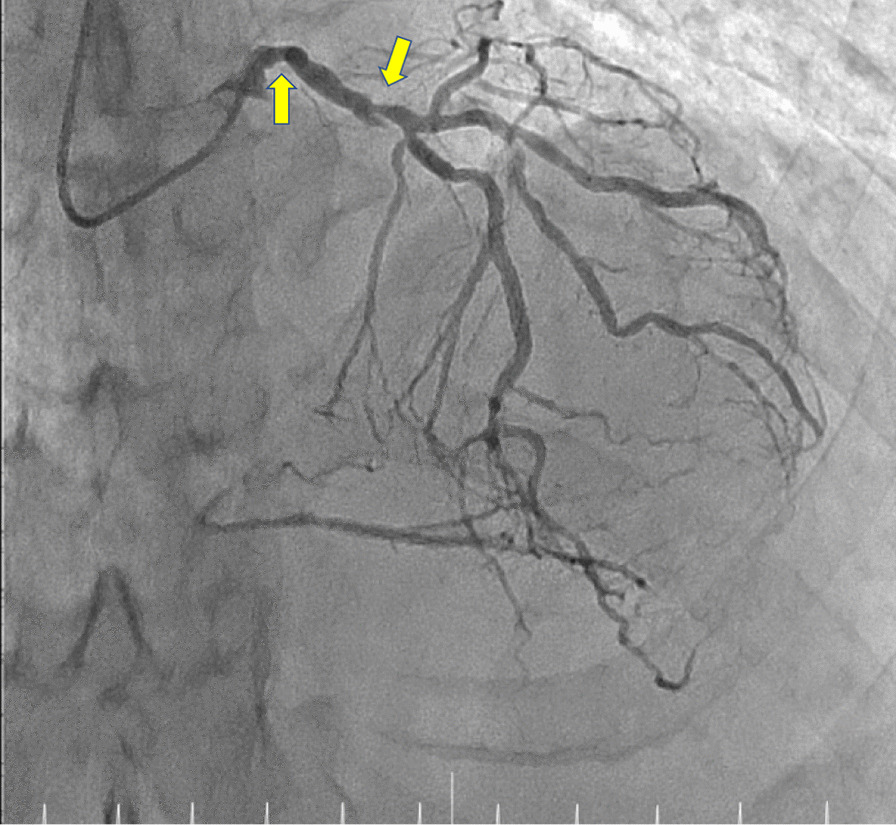
Perioperative coronary angiography demonstrating the stenosis of left main trunk (#5) and left anterior descending artery (#6) (arrows) The circumflex artery was completely occluded and the distal part of it, which was quite small, was perfused with collaterals. The right coronary artery was completely occluded and the distal part of it was perfused with collaterals

After treatment for the stroke, he was referred to our heart and vascular team because of the ASO. An angiography through the left radial artery showed total occlusion of the right common iliac artery, 50% stenosis of the left common iliac artery, and 90% stenosis of the left extra iliac artery. Coronary angiography (CAG) was simultaneously performed because he had shortness of breath during exertion. The CAG showed three-vessel disease (#1, 100%; #5, 50%; #6, 90%; #11, 100%). The circumflex artery was perfused by the collaterals, but quite small, and right coronary artery, which was filled with collaterals, supplied the posterolateral area (Fig. 1). Bilateral ITA demonstrated a good collateral pathway to both external iliac arteries.

Echocardiogram revealed that Left ventricular ejection fraction was 45% by modified Simpson method, the wall motion in the base and mid potion from the inferior to posterior lesion, and there was no significant valvular disease. Preoperative evaluation according to Euro score 2 and STS score were 1.226% and 2.09% respectively.

Our heart and vascular team concluded that CABG was recommended with bilateral ITA after a percutaneous transluminal angioplasty (PTA) because it was thought that keeping the access route for Intra-Aortic Balloon Pumping (IABP) was important and ITAs serve as good collaterals to both femoral arteries. In case of PTA failure of the right iliac artery occlusion, a concomitant procedure such as a femoro-femoral crossover bypass was planned for the CABG.

**Fig. 2:**
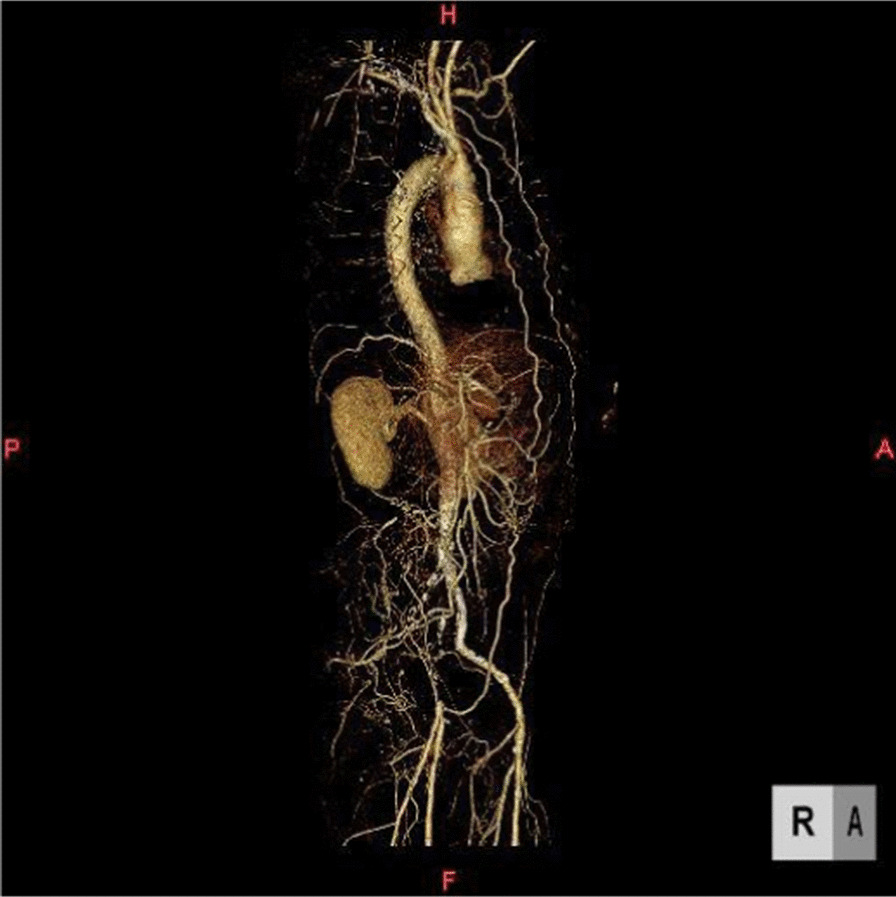
Preoperative computed tomography demonstrated that bilateral internal thoracic arteries are good collateral pathways to the external iliac arteries. Although PTA was performed to the stenosis of the left common iliac artery and the external iliac artery, the PTA to the right common iliac artery was not successful

Although the PTA to the left common iliac artery and the left extra iliac artery with stenting was successfully performed, the PTA to the occlusive lesion of the right iliac artery was not successful. (Fig. 2) Therefore, CABG with a femoro-femoral crossover bypass was performed. The left ITA was harvested, followed by the right ITA, both with a skeletonized technique. The patient underwent off-pump CABG with the left ITA, which was anastomosed to the left anterior descending artery, and the right ITA, which was anastomosed to the proximal part of the posterior descending branch. Then, the femoro-femoral crossover bypass was performed with an 8-mm ringed polytetrafluoroethylene graft. Anastomosis to the left common femoral artery in endo-to-side fashion was followed by anastomosis to the right common femoral artery in endo-to-side fashion. During the operation, the blood pressure was stable with norepinephrine (range: 0–0.06 µg/kg/min) and monitoring of the cardiac output revealed a flow of 3.3–4.3 L/min (cardiac index was 1.6–2.1 L/min/m^2^). The interval between the clamping of the right ITA and starting perfusion to the right femoral artery was 3 h and 47 min.

**Fig. 3:**
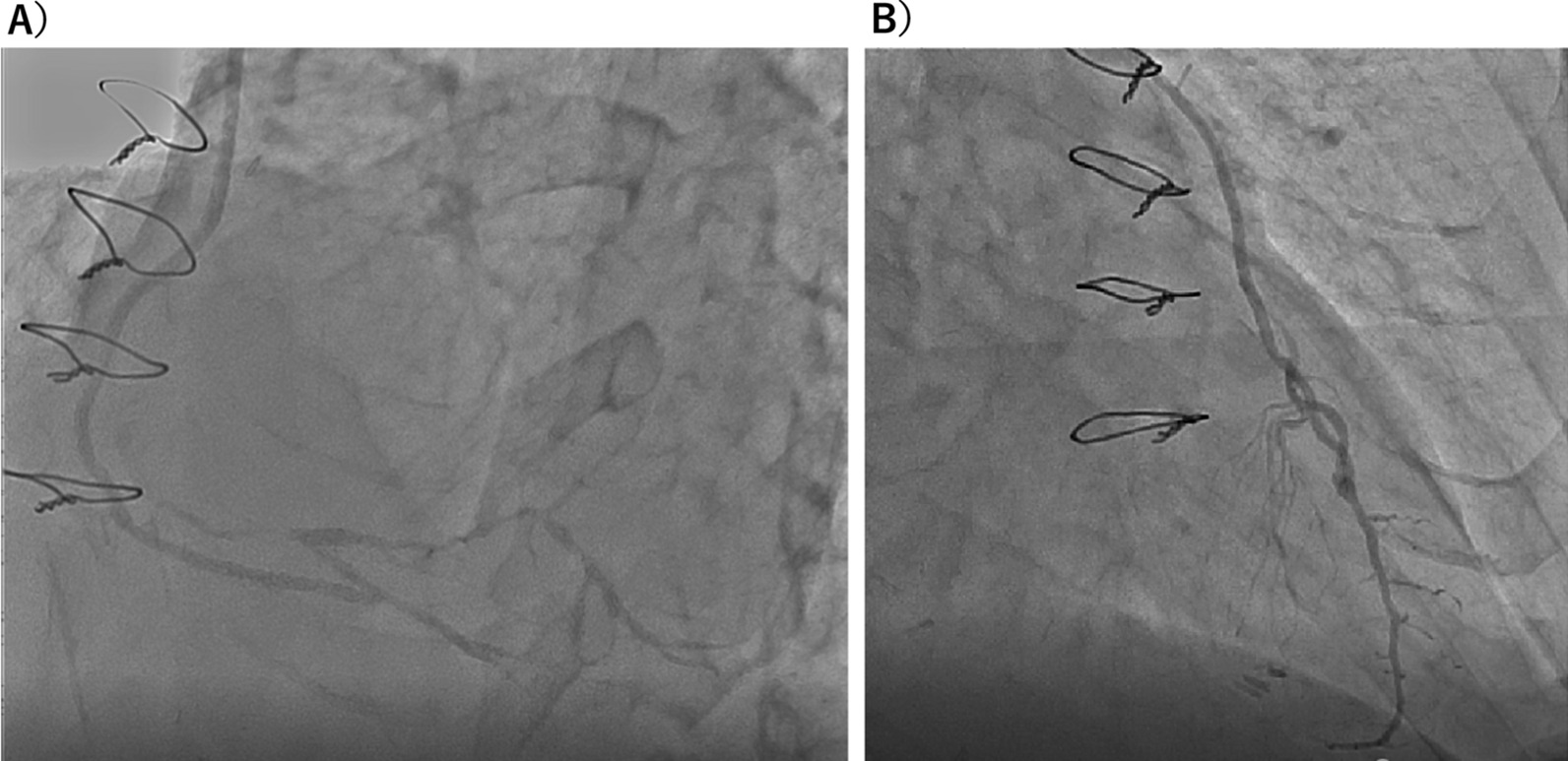
Postoperative angiogram demonstrated that right internal thoracic artery was anastomosed to the proximal part of the posterior descending branch (**A**) and left internal thoracic artery was anastomosed to the left anterior descending artery (**B**)

After the operation, the patient’s condition was stable. Two hours after the operation, he was extubated in the intensive care unit, and no significant findings in his legs were noted. He underwent a routine postoperative blood test and showed an elevated creatine kinase level (2669 U/L), which peaked out at 7177 U/Lon postoperative day 1. Subsequently, it decreased gradually to the normal range for 10 days. Postoperative angiography showed that all ITA grafts and the femoro-femoral crossover bypass were patent. (Fig. 3) The intermittent claudication symptoms improved, and the ABI was 0.95 in the right side and 0.88 in the left side. He is followed as outpatients without symptom of angina 6 months after surgery.

## Discussion and conclusions

Given the patency rate of grafts in CABG, the use of arterial grafts should be considered, especially for the bilateral ITA. On the other hand, in patients who need CABG with peripheral arterial disease, especially for iliac occlusive disease or Leriche syndrome, whose ITA could serve as an important collateral pathway to the lower extremities, the use of the ITA has been controversial because it might cause catastrophic ischemic events after surgery which result in major amputation or mortality [2, 3] With regard to the patency of grafts in CABG, there are no clear standards on how to manage such patients.

Simultaneous surgery for aortobiiliac artery bypass has been reported for cases in which ITA provides collateral circulation to the lower limbs [4, 5]. However, anatomical bypass through laparotomy, which should be used at the same time as the off-pump CABG, comes with concerns regarding its high invasiveness. To avoid such invasiveness, if possible, a preoperative approach is important for avoiding postoperative complications. Hirose et al. reported a staged operation for angina combined with aorto-iliac obstruction disease. They recommended that in patients with stable angina, surgery for aorto-iliac disease should be performed prior to CABG using ITAs because of the risk of leg ischemia [6].

Recently, the results of endovascular repair for aorto-iliac occlusive diseases such as Trans-Atlantic Inter-Society Consensus II (TASC-II) C/D lesions were improved and revealed lower perioperative morbidity and mortality compared with open surgery [7]. Additionally, unilateral repair for iliac lesions with femoro-femoral crossover bypass has been recognized as an appropriate alternative because of its lower morbidity [8].

In our case, the patient was 58 years old and had three-vessel disease with left main trunk (LMT) disease combined with symptomatic peripheral artery disease of the bilateral iliac lesions. Considering the long-term patency of bypass grafts in CABG, the hybrid operation was performed; first, performing preoperative PTA for the iliac artery; and second, performing CABG with the use of bilateral ITA. This hybrid procedure was found to be better, compared with CABG without the use of ITA or with the use of ITA combined with other grafts (e.g., radial artery or saphenous vein graft). In addition, PTA preceded the iliac lesion before the operation, but because it was not successful for the right side, we decided to perform the operation at the same time as the CABG. When femoro-femoral crossover bypass is performed after CABG, blood flow from the collateral flow to the lower limbs after the ITAs were harvested is interrupted, and there is a time lag until the blood flow to the lower limbs resumes. The influence of this interruption has not been discussed extensively. After the operation, this interruption did not appear to be a major issue, but the patient experienced an increase to a maximum creatinine kinase value of over 7000 U/L. Although we considered performing the femoro-femoral crossover bypass immediately after harvesting the bilateral ITA, we finally decided to perform it after off-pump CABG in case of an emergency of the IABP. Additionally, we thought that another collateral flow from the inferior mesenteric artery through the pelvic network could supply blood flow at the same extent during the operation. Yurdakul et.al. showed that the average flow volume and contribution of the ITA collateral pathway to lower-extremity perfusion was 66 ± 48 mL/min and 38 ± 23% in patients with aorto-iliac occlusion [9]. However, judging from the result in this case, blood flow restarted to the right lower limbs 3 h and 47 min later, so ischemia due to the effect of the ITA blockade is considered the cause of the abnormally high creatinine kinase value, although there were other collateral pathways. Considering the ischemic time to complete revascularization is important for the prevention of postoperative complications when combined operations are planned.

In this case, the patient could afford to add a PTA for bilateral iliac artery lesions prior to the CABG, but in emergency cases, it is extremely important to avoid lower limb ischemia, such as when performing simultaneous aorta–femoral bypass surgery. The use of ITA without recognizing its role as an important collateral pathway to the lower limbs could cause crisis limb ischemia [2, 3]. Because of the plaque in the patient’s ascending aorta that was detected by intraoperative direct echo, proximal anastomosis at the ascending aorta was avoided. Furthermore, the use of the radial artery such as for a Y-composite graft as left ITA with a radial artery could be an option, but due to chronic renal failure and our policy in CABG was that left ITA was anastomosed to left anterior descending artery not as a composite graft if possible.

In the approach to patients who have coronary artery disease with peripheral artery disease, especially those with iliac artery occlusion lesions with collateral circulation from the ITA, not only graft selection but also treatment strategies for peripheral lesions are considered extremely important. Hybrid operation could be an appropriate treatment option.

In conclusion, hybrid single staged coronary and lower limb artery revascularization can be safely achieved by multidisciplinary team strategies. Use of ITA for CABG in patients PVD requires careful consideration as this may be the significant collateral supply to the lower limbs.

## Data Availability

The clinical dataset used in this case report are available from the corresponding author on reasonable request.

## Abbreviations

ABI: Ankle brachial pressure index
ASO: Arteriosclerosis obliterans
CABG: Coronary artery bypass grafting
FEV1.0: Forced expiratory volume 1.0 second
IABP: Intra-aortic balloon pumping
ITA: Internal thoracic artery
LMT: Left main trunk
PTA: Percutaneous transluminal angioplasty
